# Developing ActivABLES for community-dwelling stroke survivors using the Medical Research Council framework for complex interventions

**DOI:** 10.1186/s12913-020-05198-2

**Published:** 2020-05-25

**Authors:** Steinunn A. Olafsdottir, Helga Jonsdottir, Charlotte Magnusson, Héctor Caltenco, Mikko Kytö, Laura Maye, David McGookin, Ingibjörg Bjartmarz, Solveig Asa Arnadottir, Ingibjörg Hjaltadottir, Thora B. Hafsteinsdottir

**Affiliations:** 1 School of Health Sciences, University of Iceland, Stapi v/Hringbraut, 102 Reykjavík, Iceland; 2 Faculty of Nursing, School of Health Sciences, University of Iceland, Reykjavik, Iceland; 3 Department of Design Science, Lund University, Lund, Sweden; 4 Department of Computer Science, Aalto University in Helsinki, Helsinki, Finland; 5 Department of Computer Science, Aalto University, Espoo, Finland; 6 Grensasdeild Rehabilitation, Landspitali, The National University Hospital of Iceland, Reykjavik, Iceland; 7 Department of Physical Therapy, Faculty of Medicine, School of Health Sciences, University of Iceland, Reykjavik, Iceland; 8 Emergency, Geriatrics, Rehabilitation Services, Landspitali, The National University Hospital of Iceland, Reykjavik, Iceland; 9 Julius Center for Health Sciences and Primary Care, University Medical Center Utrecht, Utrecht, The Netherlands

**Keywords:** Stroke survivors, Home-based exercise, Technical intervention

## Abstract

**Background:**

Novel technical solutions are called for to promote home-based exercise among community-dwelling stroke survivors supported by their caregivers. Lack of resources and knowledge about how to accomplish it, has been demonstrated. The objective of this study is to describe in detail the development of ActivABLES, a technical intervention to promote home-based exercise and physical activity engagement of community-dwelling stroke survivors with support from their caregivers.

**Methods:**

The technical development process of ActivABLES was guided by the Medical Research Council (MRC) framework for development and evaluation of complex interventions as well as by principles of human-centred design and co-design. The main steps included: (1) Synthesis of evidence supporting the inclusion of balance exercises, mobility and walking exercises and exercises for the upper arm; (2) Implementation of initial user studies with qualitative data collection from individual interviews with stroke survivors, and focus group interviews with caregivers and health professionals; (3) Preliminary testing of eight prototypes with seven stroke survivors and their caregivers.

**Results:**

After the preliminary testing of eight prototypes, four prototypes were not further developed whereas four prototypes were modified further. In addition, two new prototypes were developed, leaving six prototypes for further modification: 1) ActivFOAM for balance exercises, 2) WalkingSTARR to facilitate walking, 3) ActivBALL for hand exercises, 4) ActivSTICKS for upper arm exercises, and 5) ActivLAMP and 6) ActivTREE which both give visual feedback on progress of daily exercise and physical activities. ActivFOAM, ActivBALL and ActivSTICKS are all connected to a tablet where exercise instructions are given. All the exercise prototypes can be connected to ActivLAMP and ActivTREE to give feedback on how much exercise the user has done. Settings can be individualised and recommended daily time and/or repetition can easily be changed as the user progresses to higher activity levels.

**Conclusions:**

The development process of ActivABLES was guided by the principles of human-centred design, with iterative testing of future users, and by the MRC framework of complex intervention, with a repeated process of development and testing. This process resulted in six prototypes which are available for feasibility testing among a small group of community-dwelling stroke survivors.

## Background

The global incidence of stroke is increasing while the mortality rate is decreasing [1–3]. In 1990–2016, global age-standardized mortality decreased by 36.2%, leaving more people with chronic disability [3]. The impact of a stroke depends on the lesion location and the size of the affected area in the brain [4]. Studies have reported a decrease in functional outcome, an increase of functional dependence, and a lower quality of life after stroke [4, 5]. The symptoms can be relatively mild, and the stroke survivor may be independent in activities of daily living (ADLs). On the other hand, symptoms can be so severe that the stroke survivor is dependent on others for ADLs. Additionally, about one-third of stroke survivors present with depression, which significantly impacts patients’ well-being, recovery as well as their rehabilitation [6].

Various clinical practice guidelines [7–9] and systematic reviews [10–13] have summarised the evidence of positive effects of task-oriented exercise on the various outcomes of patients with stroke. Studies have shown that 30–60 min of training per day, five to seven days per week, is effective [11]. Continuation of exercise after a period of inpatient rehabilitation is important to optimise functional level [10, 12, 14, 15] and exercise and physical activity should be a lifelong process for stroke survivors [16]. Strong evidence exists for physical therapy interventions favouring intensive highly repetitive, task-oriented and task-specific exercise in all phases after stroke [12, 17]. However, community-dwelling stroke survivors only receive a limited amount of outpatient exercise and physical activity after inpatient rehabilitation. In four European countries, physical therapy was the most frequently used follow-up health service after inpatient rehabilitation, apart from medical care provided by a general practitioner [18]. Physical therapy services may only be available for a limited amount of time per week, which does not fulfil the need for daily exercise and physical activity. Therefore, community-dwelling stroke survivors need to be motivated to continue with home-based exercise and engage in physical activity without the constant supervision of professionals. For that reason, finding ways to motivate stroke survivors to engage in home-based exercise and physical activity is highly important.

Stroke survivors often have little motivation and confidence to continue with home-based exercise on their own after hospitalisation or inpatient rehabilitation [19, 20]. Lack of motivation and understanding about how to incorporate daily activities into an exercise plan, have been reported as reasons for limited unsupervised exercise adherence of stroke survivors [21]. A systematic review synthesised the evidence from six studies, exploring perceived barriers and motivators to physical activity after stroke, and showed that lack of motivation was a barrier to physical activity, in addition to environmental factors and health concerns [20]. Another systematic review focused on the designing of rehabilitation games and explored stroke survivors’ motivation in rehabilitation. Factors positively influencing stroke survivors’ level of motivation included social and emotional support from family members, the patient-therapist relationship, goal setting and music [22]. When designing ways to promote exercise and facilitate physical activity, it is important to understand what factors can motivate and hinder stroke survivors in exercise and physical activity.

The literature shows that informal caregivers (hereafter referred to as caregivers), who are often family members, express willingness and are often able to support stroke survivors with home-based exercise, resulting in the stroke survivors acquiring improved physical and mental function [12, 23–25]. Still, they often lack knowledge about what and how to do it and need more professional support and/or supervision to feel secure supporting the family member after stroke [26, 27]. Therefore, it is important to find ways and resources to support them in encouraging home-based exercise and increased physical activity for the stroke survivors.

Studies have reported good adherence of community-dwelling stroke survivors to exercise and perform physical activity when using technical applications in their homes to support these activities [28–30]. Technical interventions and applications that can be used for exercise and physical activity are increasingly being researched for different groups, including stroke survivors. Tangible interaction offers significant potential benefits, creating tangible user interfaces (TUIs) that are easy to handle for persons with cognitive or motor impairments [31]. Moreover, there are indications that the use of technology can motivate stroke survivors to engage in home-based exercise and physical activity [32], and motivational feedback seems to be the most important factor in technical solutions [33]. Technical interventions can offer repetitive and challenging exercises which are necessary for brain plasticity and motor learning [34]. The results from reviews of use of technical interventions have shown functional improvements [35, 36]. Some studies have investigated exercise in virtual reality [28, 35, 37, 38] and the use of video games, such as Nintendo Wii [36, 39–41] and Microsoft Kinect [42, 43] on which motion-controlled games may be played [22, 29, 32]. Games played through technical applications can motivate stroke survivors to participate in home-based exercise [35, 44, 45] and they are effective in improving balance and independence [46]. A systematic review showed that it is important that stroke rehabilitation games combine mental support, motivation and accessible interfaces in order to have a positive impact on participation in exercise and physical activity. Empowering stroke survivors to take charge of their own rehabilitation was important to initiate playing games and exercising [32].

The purpose of this paper is to report on the development of ActivABLES, a modular technical intervention built of multiple exchangeable components, to allow its thorough review and replication. The aim of the ActivABLES intervention is to motivate and support community-dwelling stroke survivors with home-based exercise to increase physical activity with support from their caregivers, and under the supervision of a physical therapist or other rehabilitation professionals. Our research question is: How can a tangible intervention, aiming to increase exercise and physical activity for community-dwelling stroke survivors, be developed with the involvement of future users?

## Methods

The design was based on the Medical Research Council Framework (MRC), human-centred design and co-design. A three-step procedure was used for the development of ActivABLES which included: (1) identifying the evidence and outcomes where we used the findings from earlier systematic reviews, (2) implementation of an initial user study and iterative tests which included qualitative individual and focus group interviews with stroke survivors, caregivers and professionals, and (3) preliminary testing where each prototype was tested in the home of seven stroke survivors for a few hours. The study was approved by the National Ethics Committee of Iceland (Ref. VSNb2015110001/03.01), the Regional Ethics Committee in Lund, Sweden (dnr 2015/678) and the City of Helsinki, Finland (HEL 2016–002570). The study was conducted between September 2015 and March 2018 in accordance with the principles of the Declaration of Helsinki, and all participants signed an informed consent for participation.

### The Medical Research Council framework

**Fig. 1:**
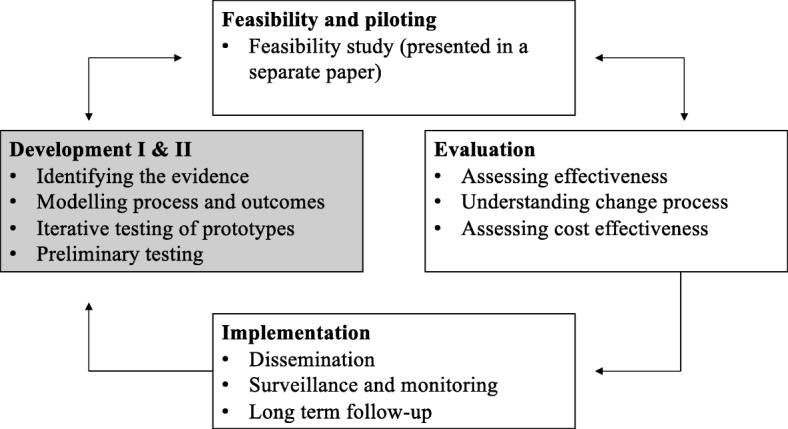
Medical Research Council framework for ActivABLES

The MRC framework for development of complex interventions was used to guide the development of the ActivABLES as a healthcare intervention. The MRC framework defines interventions that contain several interacting components as complex interventions and provides guidance for their development [47] (Fig. 1). The framework describes the process of development, which includes four phases; (i) Development, (ii) Feasibility and piloting, (iii) Evaluation and (iv) Implementation. These phases do not have a linear sequence and each one can affect the others. In this paper, we report on the first phase of the framework which includes the development of ActivABLES. We used the first two phases of the framework. The Criteria for Reporting the Development and Evaluation of Complex Interventions in Healthcare (CReDECI 2) was used to report the phases of the development process [48].

### Human-centred design and co-design

The technical development process of ActivABLES was guided by the principles of human-centred design (HCD) (ISO 9241-210:2010) and methods of co-design. HCD is a management framework that develops solutions by involving the human perspective in all steps of the problem-solving process [49]. Co-design assumes that individuals of equal cognitive and physical abilities participate in the development process [50]. This design is often used when interactive technologies are being developed. During the development process and prior to the feasibility study conducted in Iceland, small technical tests were performed iteratively in Sweden and Finland. These tests involved stroke survivors, caregivers and health professionals, and included the testing of several aspects of the prototypes, such as user interface, usability, etc. The technical and design process has been described in previous papers: the initial studies and user requirements [51], the balance part of the technical system [52], the development of the arm/hand tools [53] and the app design [54].

### The ActivABLES team

**Fig. 2:**
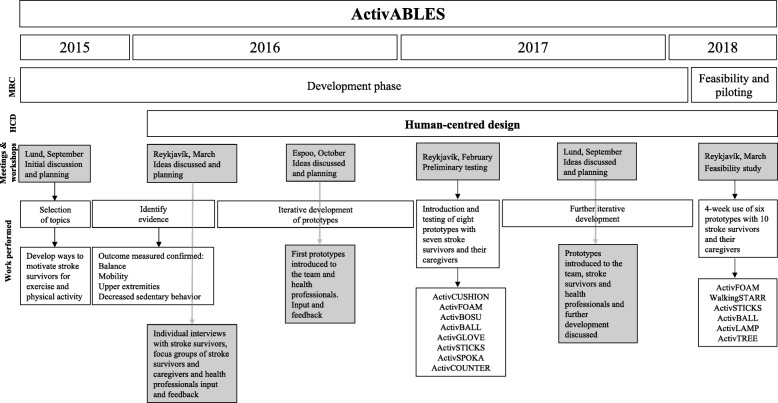
Timeline and process of the development of ActivABLES

The ActivABLES research team includes multi-disciplinary researchers from (i) Iceland: five nurse scientists (TBH, HJ, IB, IH, EP) and two physical therapist scientists (SAO, SAA) with extensive experience in stroke rehabilitation and research; (ii) Sweden: two design sciences engineers (CM, HC); and (iii) Finland: three computer scientists (DM, MK, LM) and one computer scientist student (WB), all with experience in the development of technical interventions in healthcare. Throughout the development process, the team had bi-weekly Skype meetings and six cross-country meetings where the research team discussed the progress of the development and the research work (Fig. 2).

### Development process of ActivABLES

The development of ActivABLES involved the three following steps (Fig. 2):

#### Step 1. Identifying the evidence and outcomes

We identified the evidence base for effective exercise interventions and important outcomes for stroke survivors. The findings of systematic reviews showed the importance of augmenting exercise and physical activities among stroke survivors [10, 15, 55]. Physical inactivity and sedentary behaviour are significant considerations at all stages after stroke (acute, subacute and chronic) and seem to increase from the subacute state to the chronic stage [56, 57]. Many stroke survivors do not continue with training and become physically inactive following inpatient rehabilitation [55, 57], often due to depression or lack of motivation [20, 22].

The following evidence from systematic reviews was used to identify the outcome measures for the ActivABLES intervention:

- Balance: Balance impairments are very common in stroke survivors, affect general mobility and walking ability [58], and increase the risk for falls. Studies have shown that 33–48% of stroke survivors fall at least once within the first year after their stroke [59]. Balance exercises can result in improved function in all post-stroke phases [12, 25]. There is strong evidence that balance can be improved with exercise, including using technical applications [25]. Balance exercises with visual or auditory feedback can have significant effects on improving the balance of chronic stroke survivors, especially those with mild to moderate impairments [12].
- Mobility: Mobility is defined as “the ability to move in one’s environment with ease and without restriction” [60] and includes the ability to stand up/sit down and walk. There is strong evidence that gait exercise significantly improves the mobility of stroke survivors in all phases after stroke [12, 25]. Task-oriented exercise and visual and auditory feedback are especially recommended as key factors for improving mobility [61].
- Upper extremity: Impairments of the upper extremities are common in stroke survivors and can cause difficulties in different activities of daily living such as eating, dressing and washing [62]. Various reviews have emphasised the importance of exercise for the upper extremities, especially for stroke survivors with mild to moderate impairments, and they can benefit from exercise emphasising task-specific repetitions, which is a key factor in motor learning [12, 17, 25, 34].
- Motivation: Motivation for exercise and physical activity is often lacking after a stroke [20] and about one-third of stroke survivors deal with depression, which can affect motivation [6]. Motivational interventions, including internet-based programmes and reinforcement strategies, can increase adherence to exercise [63]. Feedback can motivate stroke survivors to engage in exercise and physical activity, and both visual and audio feedback can motivate stroke survivors to continue with exercise. Support from caregivers and health professionals is also important [20, 64].

#### Step 2. Initial user study and iterative tests

We involved stroke survivors with a mild to moderate level of physical disability (Modified Rankin Scale 2–4), informal caregivers, rehabilitation professionals and other stakeholders in the whole development process to gain their feedback on the development. An initial user study was conducted in Iceland. This included a qualitative focus group interview and 10 individual interviews with stroke survivors. The qualitative interviews were thematically analysed according to Brown & Clark (2006), resulting in three identified themes: managing the challenges of impairment, long-term challenges of everyday life and framing exercise within the context of everyday life. These findings emphasised the importance of exploring innovative ways of using technology to empower stroke survivors to tackle challenges and be responsible in their daily activities, and to motivate them to engage in home-based exercise and physical activity. The results of this study will be published in a separate paper (Hafsteinsdottir et al. 2020/work in progress). In addition, ideas and prototypes were introduced and discussed with stroke survivors, caregivers and professionals at the on-site team meetings, and iterative tests were performed in Sweden and Finland to test the design and technical systems of the prototypes [51–54]. Workshops were also held for stroke survivors, caregivers and rehabilitation professionals during the development process, where different prototypes were presented to solicit feedback and input on usability.

#### Step 3. Preliminary testing of prototypes

In the context of development according to the MRC framework, a preliminary testing of eight ActivABLES prototypes was conducted in February 2017 (Fig. 2). This preliminary testing aimed to investigate how the prototypes could be used by stroke survivors in their home environment, and to gain feedback on the development and feasibility of ActivABLES prototypes. The testing took place in the stroke survivors’ homes and lasted for 1–2 h. Each prototype was tested until the stroke survivors had tried all the exercise and activity possibilities each prototype included. Observations were made, and participant-researcher interactions were video-recorded. During the observations, we asked questions and received feedback on each prototype and one of the researchers filled in a form with comments on each prototype (Additional file 1: Appendix I). These comments were used to improve and further develop the prototypes, along with input from the technical team. Following the testing, the researchers conducted semi-structured interviews separately with each stroke survivor and his/her caregiver, using interview guides (Additional file 1: Appendix II). Additional questions were asked to gain feedback concerning experiences, meaning, technical elements of the prototype (background, light, sound, objects etc.).

A purposive sample included seven community-dwelling stroke survivors ≥18 years, with a mild to moderate level of physical disability (Modified Rankin Scale 2–4) and their caregivers. The age range of the stroke survivors (four women and three men) participating was 31–76 years and their strokes had occurred from 9 months to 22 years previously. Functional outcome measures were carried out to provide a thorough description of the stroke survivors participating (Table 1). Six caregivers participated, three men and three women, in the age range of 53–75 years. All of them were spouses of the stroke survivors, four were employed and two were retired.

**Table 1:** Characteristics of the participants in the preliminary testing

Stroke survivors	Age	Time since stroke	Side of hemiparesis	Walking device inside	BBS^a^	BBT^b^	ABC^c^
SS-1	31	19 months	left	no	56	x	76.3
SS-2	60	4 years	left	no	37	x	38.1
SS-3	62	9 months	left	no	47	x	51.4
SS-4	63	22 years	left	no	43	x	66
SS-5	66	2 years	right	no	33	53	65
SS-6	72	4 years	left	yes. a cane	43	6	73.1
SS-7	76	9 years	left	yes, a cane	37	12	29.4

*SS* stroke survivor

^a^Berg Balance Scale, score 0-56 where lower score indicates more balance impairments

^b^Box and Block Test, number of blocks moved between boxes in one minute. X presents those who were not able to use their affected hand

^c^Activities-Specific Balance Confidence Scale assesses self-efficacy in different activities, score 0-100 where 0 represents no confidence and 100 represents complete confidence

Eight ActivABLES prototypes were introduced in the preliminary testing, namely: ActivCUSHION, ActivFOAM, ActivBOSU, ActivBALL, ActivGLOVE, ActivSTICKS, ActivSPOKA and ActivLAMP. The findings of the preliminary testing showed that four prototypes were found to be relevant for further development for community-dwelling stroke survivors with mild to moderate impairments: ActivFOAM, ActivBALL, ActivSTICKS and ActivLAMP. During the preliminary testing, the stroke survivors and their caregivers indicated that there was a need for an application to stimulate and detect more general activity like walking. This result was further supported by a workshop carried out in the EU project STARR at the Stroke Organisation in Bromsgrove, UK [65]. Therefore, as a joint effort with this EU project, WalkingSTARR was developed, which is an application for the iPhone with a step-counter and games to encourage walking. In addition, ActivTREE was developed as an application providing feedback on more than one exercise/physical activity (Table 2).

**Table 2:** Prototypes of ActivABLES

	Preliminary testing	Feedback during observations	Revisions of the prototype
**ActivFOAM**	The foam was connected to a tablet where the users could see how their weight was distributed on the mat, get audio feedback and play one game (The bomb). The user could see on the screen when weight was shifted from 1 foot to the other.	“It is very convenient to stand on this and see how I am standing. It gives you comments on how you are standing”.	Two games were added as ways to practice balance. Also, there were possibilities to use different music to encourage weight shifting and stepping one the mat.
**WalkingSTARR**	Not yet developed.		After the preliminary testing it was decided to develop an iPhone application to encourage walking.
**ActivBALL**	The ball was introduced as a mouse for a computer when browsing Google Street View, and online magazines and for basic internet browsers, and to play basic games such as Tic-Tac-Toe. It could also be used as a tool for squeezing (or do other exercisesfor the hand/wrist) to “earn” a series from television/Netflix.	“I think it could work as a mouse - it would be a more suitable movement [for the hand]”.	Due to lack of time, it was not possible to develop these possibilities further prior to the feasibility study. Therefore, the exercises were repetitive movements with the recommended number of daily exercises seen on the tablet. A counter for the exercises was visible on the tablet.
**ActivSTICKS**	The sticks were introduced as a tool to use to browse Google Street View. The idea was to have a double-arm tool to use for “wandering around” on Google streets.	The users found it difficult to handle the sticks. Although the idea was new to the users, it was decided to develop it further.	Due to lack of time, it was not possible to develop these possibilities further prior to the feasibility study. Therefore, the exercises were repetitive movements with the recommended number of daily exercises seen on the tablet. A counter for the exercises was visible on the tablet.
**ActivLAMP**	The light gave feedback on how long the users had been exercising.	“I think it is rewarding to see the light strip become progressively more lit up”.	ActivLAMP was further developed into a single light strip in a stained glass cylinder that lit up as the user used one ActivABLES tool.
**ActivTREE**	Not yet developed.	“It would be good to have something that gives an overview of the exercises”	After the preliminary testing, it was decided that it was necessary to provide feedback on multiple activities at the same time.

ActivCUSHION and ActivBOSU were not considered to be appropriate for community-dwelling stroke survivors with slight to moderate symptoms. ActivCUSHION is a thin cushion that can be put on a seat and then the stroke survivor sits on it. Pressure sensors pick up the weight and give individually tailored visual and sound feedback on posture while seated and for example warning if the stroke survivor is leaning more towards one side. It was not challenging enough for the stroke survivors who participated since they generally had good sitting balance. Still, the stroke survivors and researchers agreed that stroke survivors with impaired sitting balance could benefit from using it. ActivBOSU is a half ball, with an unstable base, and can be used for balance and posture exercise. It was considered to be too difficult and not safe to use for balance exercise at home, since it is quite challenging to stand on the soft and unstable surface of ActivBOSU and doing so would increase the risk of falling while doing the exercises. However, it was deemed to be fitting for supervised use by stroke survivors with good active balance. ActivSPOKA is a little lamp which lights up to remind the stroke survivor to exercise and/or to give feedback when the daily recommended exercises and physical activity are finished. Due to similarities and redundancies with ActivLAMP it was excluded from further development. ActivGLOVE gave promising results, with possibilities of extension and flexion extension movements of the fingers, but it was too difficult for the stroke survivors to put it on and further design was needed to make it more suitable. Therefore, four of the prototypes were excluded after the preliminary testing: ActivCUSHION, ActivBOSU, ActivSPOKA and ActivGLOVE (Table 3).

**Table 3:** Excluded prototypes after the prelimary testing

	Preliminary testing	Feedback during observations	Reason for exclusion
**ActivCUSHION**	The thin cushion was put on a chair and could give feedback on weight bearing in sitting, as it was connected to a tablet. The idea of different feedback was discussed.	“I would sit up straight, for example in front of the television or when working by the kitchen table”.	We thought that only very few users with mild or moderate impairments would be in need of this kind of tool. Therefore, it was decided not to develop it further at this point. Still, we got some ideas on different feedback, i.e. vibration that would be more private than a light or a sound.
**ActivBOSU**	Only one user who had hardly any balance difficulties, was able to try ActivBOSU.		It was decided that ActivBOSU was too difficult for users to use safely in their homes.
**ActivGLOVE**	The glove had visual and audio feedback with the purpose to extend the fingers. The finger lit gradually when the finger was extended or played a sound when it was fully extended.	“The glove needs to be a mitten or not for each finger”. “It would be a good idea to have a specific sound for the movement of each finger”.	It was hard to put the glove on and it was decided another version was needed which would be more open and easier to put on. This version would fit all hand sizes. Further development of the glove turned out to be quite complex and needed extensive expertise. Therefore, it was decided not to develop it further at this point.
**ActivSPOKA**	The light gave feedback on how long the users had been exercising.	“I see the purpose of this one, as a reward thing, I also think it’s just fun”. “You could have it red or green, depending on how you are doing”.	Due to similarities and redundancies with ActivLAMP and the greater flexibility of ActivLAMP, ActivSPOKA was not further developed.

**Fig. 3:**
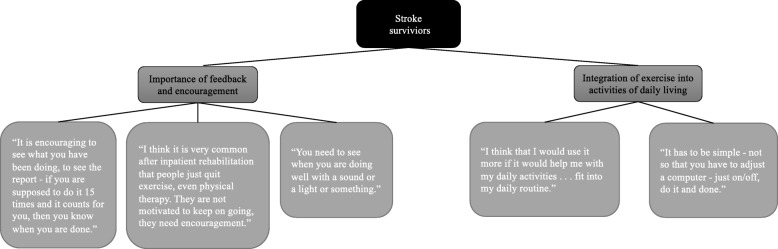
Thematic analysis of interviews with stroke survivors

**Fig. 4:**
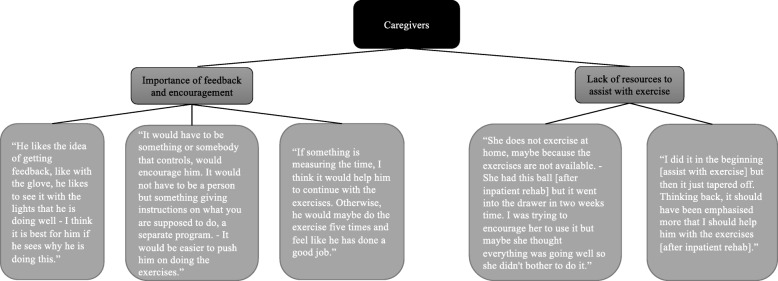
Thematic analysis of interviews with caregivers

The data from the interviews with the stroke survivors and their caregivers were analysed individually using thematic analysis (Brown and Clark, 2006). Two themes were identified for each group: *Importance of feedback and encouragement* and *Integration of exercise into activities of daily living* (Fig. 3) for the stroke survivors and *Importance of feedback and encouragement* and *Lack of resources to assist with exercise* for the caregivers (Fig. 4)*.* Based on these findings, the prototypes were further developed and adapted to the needs of the stroke survivors and their caregivers. The idea of ActivABLES was to give stroke survivors and their caregivers resources to use for exercise and physical activity. The prototypes were made small to make them easy to use in homes and accessible in daily life. As requested, some form of feedback mechanism was included into all of the prototypes.

## Results

The development process resulted in the six following prototypes relevant for community-dwelling stroke survivors with slight to moderate impairments: ActivFOAM, WalkingSTARR, ActivBALL, ActivSTICKS which are exercise prototypes and ActivLAMP and ActivTREE which give visual feedback on the amount of exercise done.

**Fig. 5:**
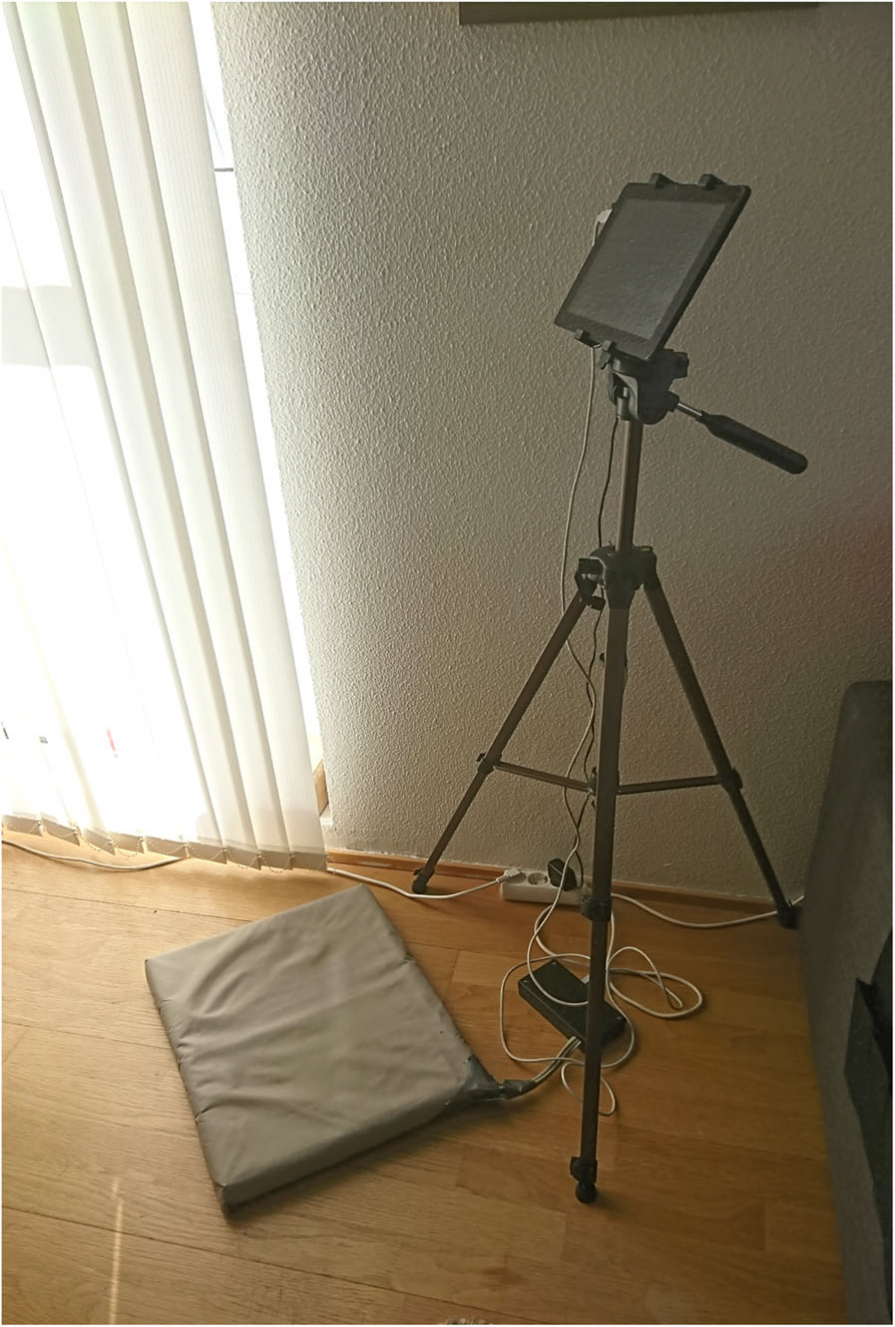
Setup for ActivFOAM

**Fig. 6:**
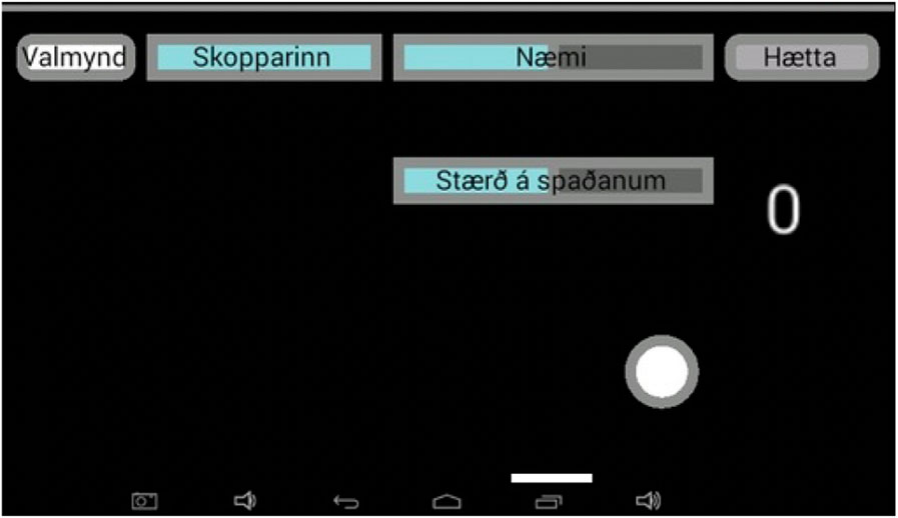
Screenshot of *Pong*

**Fig. 7:**
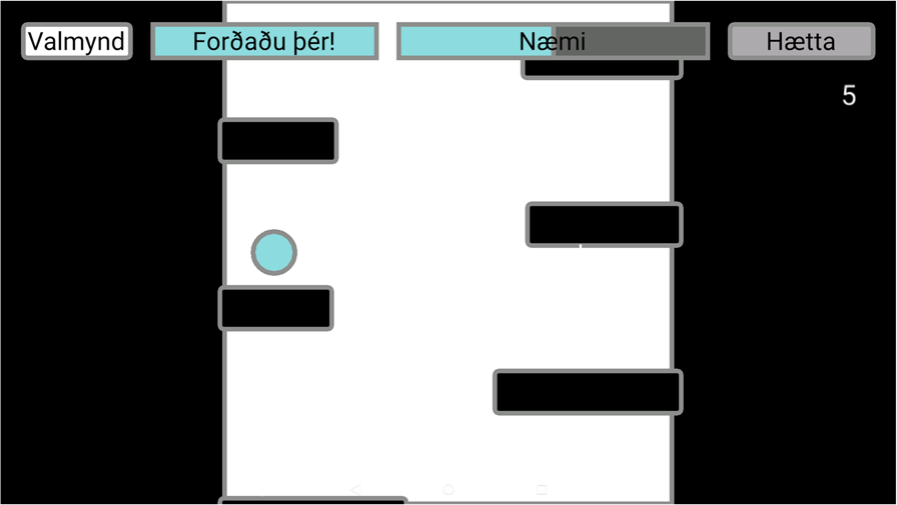
Screenshot of *Escape*

**Fig. 8:**
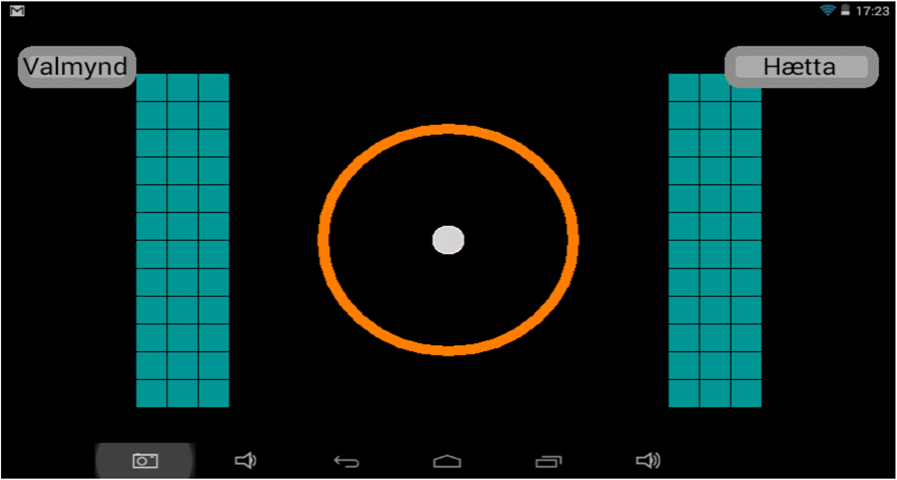
Screenshot of *Bomb*

ActivFOAM is a soft balance mat with pressure sensors that give individually tailored visual and sound feedback on weight shifting and centre of mass while standing [52]. The mat is connected to a tablet which is positioned in front of the user (Fig. 5). Three interactive games and different types of audio feedback can be selected from the tablet and used for exercising:

(i) *Pong* for reactive balance, where the user moves a paddle by shifting the amount of weight on each foot to hit a ball which comes at different speeds from an unknown direction (Fig. 6). The user has to shift more weight to the other foot to make the paddle move. The size of the paddle can be adjusted: smaller paddles make the game more difficult. The user collects a point each time he/she hits the ball.
(ii) *Escape* for proactive balance where the user moves a ball, by putting more weight onto 1 foot to avoid barriers which are in the way (Fig. 7). The user collects a point for each barrier he/she escapes.
(iii) *The bomb* for proactive balance, where the user moves a ball in and out of a circle. The ball is moved outside the circle by putting more weight onto 1 foot, as much as the user is able to, and then back into the circle by adjusting the weight onto both feet. The ball needs to be back in the circle before audio feedback indicates a bomb explosion (Fig. 8).
(iv) More possibilities include use of different types of audio feedback like jazz, samba and guitar tones while looking at a screen showing how much weight is being put on each leg.

**Fig. 9:**
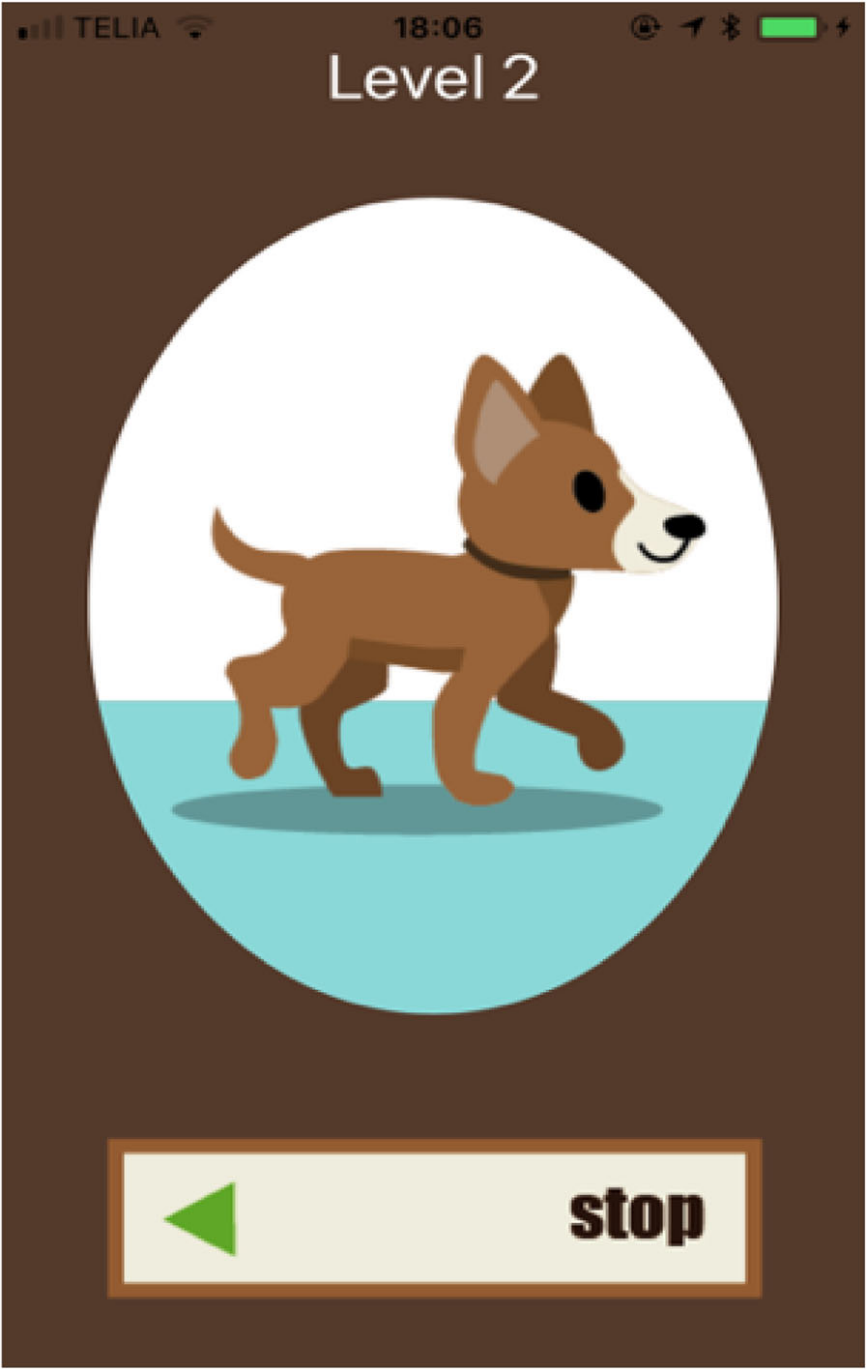
The ellipse fills up with blue in WalkingSTARR

WalkingSTARR is an iPhone application that includes a step counter which records the daily steps taken and walking time [54]. Daily recommendations for the number of steps to take can be individualised in the app for each user. The idea is to mimic taking the dog out for a walk and the app “barks” randomly during the day to remind the user to go for a walk. The app also includes a few optional tasks which involve having to stop to let the dog pee by a tree and eat food from a bowl. The user needs to point the iPhone in certain directions to find the tree and the bowl, which are visible on the iPhone. These tasks require some motor control where the user has to initiate and stop walking to meet the dog’s needs. The user might also have to turn in order to point the iPhone into the right direction. These tasks are supposed to be motivating as the user collects stars when each task is completed. The visual feedback can be seen in Fig. 9 where the ellipse gradually fills up with colour as the daily recommendations are met.

**Fig. 10:**
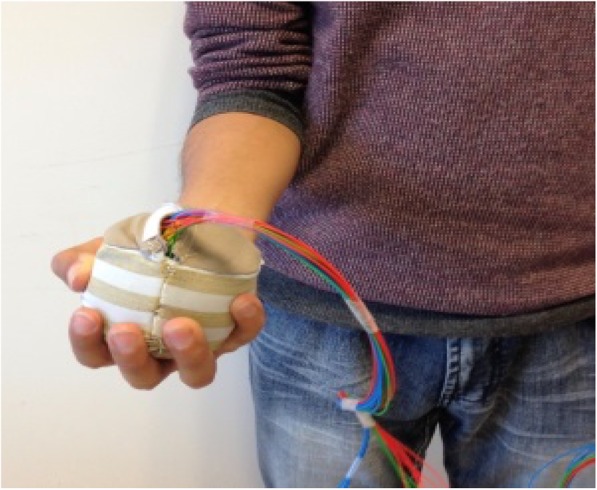
Using ActivBALL for exercising supination/pronation of the hand

ActivBALL is a soft ball which is intended to improve motor control of the forearm and upper arm, and grip strength. The ball is connected to a tablet which is positioned in front of the user and can be pre-programmed for individually tailored sets of exercises. The ball can be used to exercise the following movements: 1) Forearm pronation and supination (Fig. 10), 2) Dorsiflexion and palmar flexion of the wrist, 3) Flexion and extension of the fingers while squeezing, and 4) External and internal rotation of the shoulder. The range of motion and pressure detected while squeezing can be adjusted for each user. While exercising using ActivBALL, the user follows instructions on the tablet about the number of repetitions and sets, both of which can be individualised for each user.

**Fig. 11:**
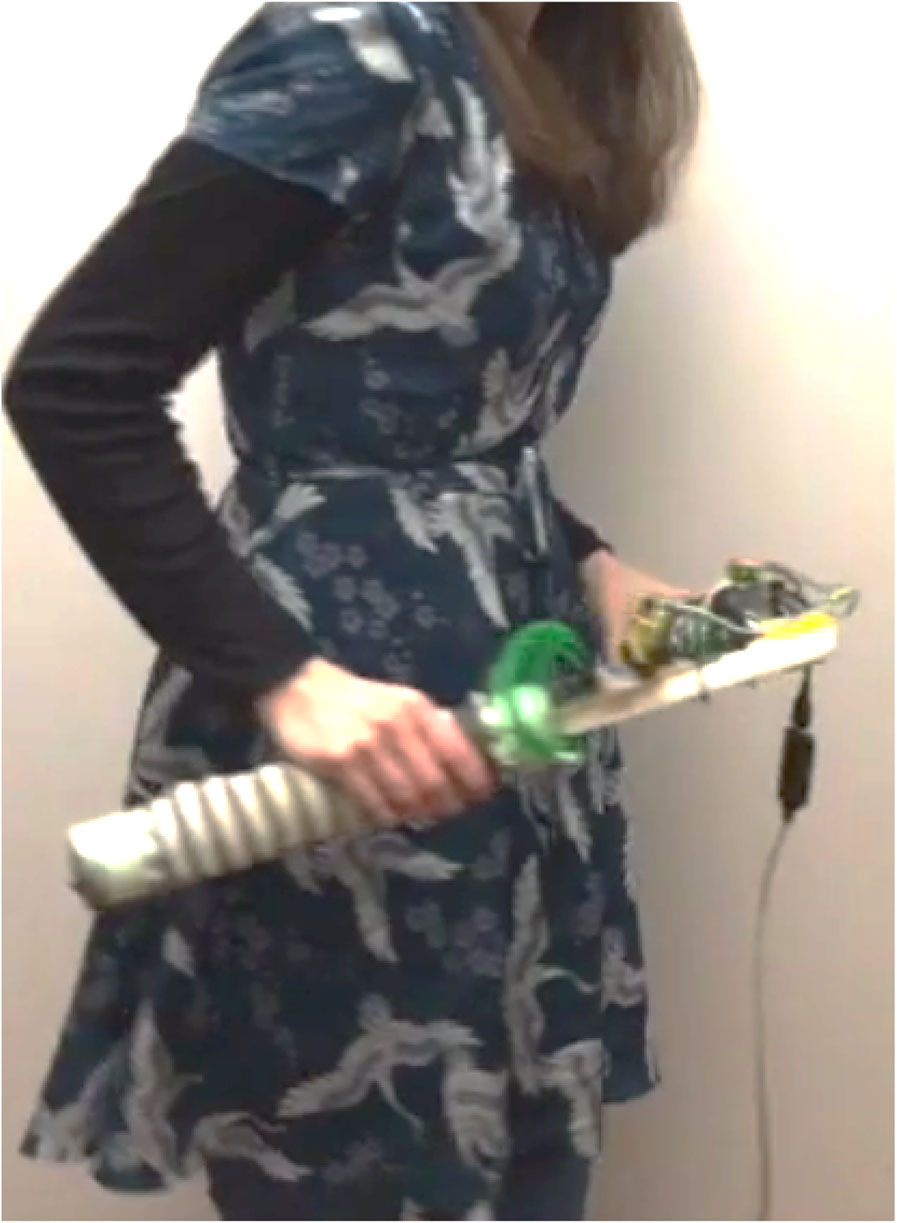
Using ActivSTICKS for rotation of upper body

ActivSTICKS consists of two plastic sticks which are linked together forming an angle from 0° to 180° [53]. The sticks are connected to a tablet which is positioned in front of the user and can be pre-programmed for individually tailored sets of exercises. The sticks can be used to perform the following movements: 1) Abduction and adduction of the shoulder, 2) Flexion of the shoulder, 3) Elbow flexion and extension along with coordination of the left and right arms while doing “scissors”, and 4) Rotation of the upper body (Fig. 11). The range of motion as well as the resistance to the movement can be adjusted for each user. While exercising using ActivSTICKS, the user follows instructions on the tablet about the number of repetitions and sets, both of which can be individualised for each user.

ActivLAMP and ActivTREE give visual feedback on the stroke survivor’s daily progress by gradually lighting to indicate the proportion of exercises completed. The more exercises done or steps taken, the more the ActivLAMP/ActivTREE lights up. ActivLAMP and ActivTREE reset every day at midnight. Settings can be individualised for each user and the recommended use per day and/or number of repetitions can easily be changed as the user further progresses to higher activity levels. A rehabilitation professional gives instructions on which prototype to use and for how long the user should exercise based on a baseline functional assessment and preferences for each stroke survivor.

## Discussion

In this paper we provide a detailed report of the three-step development process of ActivABLES for community-dwelling stroke survivors and their caregivers to allow for a thorough review and replication of the process. The development process resulted in six prototypes: four exercise prototypes which are ActivFOAM, WalkingSTARR, ActivBALL and ActivSTICKS, along with ActivLAMP and ActivTREE, which give visual feedback on the amount of exercise done. Three of the exercise prototypes and the two feedback prototypes were connected to a tablet but WalkingSTARR was only developed as an application for iPhone. Digital servers store data about all uses of the prototypes. The tangible prototypes do not take up much space and can easily be used in a small environment, such as a small room. In this respect they are different from many other technical solutions, such as Wii and Kinect, where a television or a bigger screen is needed. ActivABLES also offer different activities aimed at different functional outcomes but do not focus solely on one single exercise or activity. The challenges in each exercise and physical activity can be individualized for each user. ActivABLES is specially developed for community-dwelling stroke survivors, since these stroke survivors and their caregivers have called for more opportunities for exercise and physical activity in their own home [19, 66]. The results of the three steps in the development process support our ideas that ActivABLES is relevant for community-dwelling stroke survivors with mild to moderate symptoms. The evidence found in the literature shows what kind of exercise and physical activity are relevant for community-dwelling stroke survivors. The initial user study gave us an idea about where to put the emphasis in the development, and the iterative technical testing during the development made the prototypes useable in the preliminary testing. The results of the preliminary testing gave positive feedback for further development and preparation for the feasibility study which is presented in another submitted paper (Olafsdottir et al. 2020/work in progress).

Much innovative research and many interventions are ongoing, and they often lack a thorough description, which is important to improve replicability. The CReDECI 2 guidelines for reporting of the development of complex interventions [48] proved useful to report the first and second stages of the development of ActivABLES in order to ensure the quality of transparent reporting of this complex intervention. Also, the MRC framework provides guidance for development, of the ActivABLES intervention as a complex intervention. The reporting of the feasibility testing of the ActivABLES is given in another paper (Olafsdottir et al. 2020/work in progress) and studies on the other phases of the MRC framework, including the evaluation and the implementation, are still to be done (Fig. 1). The design of the study, using a human-centred approach and co-design in which stroke survivors, caregivers and rehabilitation professionals participated, is highly important, with the potential future users involved in every step of the development process. A key element in the process has been to involve not only potential future users, but also the context of potential future situations of use, the stroke survivors’ homes.

The six ActivABLES prototypes developed include: ActivFOAM for balance exercise, WalkingSTARR for encouraging walking, ActivBALL for hand exercises, ActivSTICKS for arm exercises and ActivLAMP and ActivTREE for feedback on exercise.

ActivABLES seems to be very suitable for helping caregivers to support the stroke survivors in exercising at home. This is important as studies have shown that caregiver-mediated home-based exercise can give good functional results [23, 67] and can have a positive impact on anxiety and depression of both the stroke survivor and the caregiver [24]. In addition, caregivers are willing to be more involved in the rehabilitation process at home if they have more information and knowledge about how they can support and motivate their stroke survivor to exercise and be more physically active [66, 68, 69].

Other studies using interactive games, similar to games with ActivFOAM, have shown promising results regarding adherence acceptability and safety [29]. ActivABLES aims to motivate users and make home-based exercise and physical activities more fun and less tedious with more variety in exercise and training options for community-dwelling stroke survivors with slight to moderate activity limitations. ActivABLES could be a resource for physical therapists to motivate community-dwelling stroke survivors to engage and continue with home-based exercise and physical activities after inpatient rehabilitation. Further benefits of an intervention like ActivABLES may include less need for inpatient healthcare services and possible earlier discharge from hospital or inpatient rehabilitation, resulting in lower healthcare costs and other economic benefits [24, 70]. More research is needed with larger samples of community-dwelling stroke survivors and caregivers.

The main limitations of this study include technical problems, which are inherent when using experimental prototypes that are primitive and fragile and need to be delicately handled. In the development process, the technicians were involved at all times so they could solve the emerging problems. Another limitation is the small sample of participants. Among the strengths of this study are (i) the use of theoretical underpinnings, as we followed the MRC-model for complex interventions, (ii) the human-centred design which gives the researchers a thorough understanding and inputs from future users, including stroke survivors, caregivers and the multi-disciplinary team working on the idea, and (iii) the evidence-based approach, which brings out knowledge about ways to promote home-based exercise and physical activity of community-dwelling stroke survivors.

ActivABLES has the potential to be a good resource for healthcare professionals and the healthcare system to follow up on community-dwelling stroke survivors. The healthcare system is unable to provide daily support for those who need encouragement and/or support with physical activity. Community-dwelling stroke survivors need to increase their health-promoting physical activity, preferably in their own environment, with support from their caregivers and instructions from rehabilitation professionals. ActivABLES seems to be very suitable to support community-dwelling stroke survivors in exercising at home.

## Conclusion

ActivABLES is promising technical equipment aiming to support community-dwelling stroke survivors when engaging in home-based exercise and health-promoting physical activities with support from caregivers. Community-dwelling stroke survivors, caregivers and rehabilitation professionals were involved in the whole development process. ActivABLES integrates Tangible User Interfaces into the everyday activities of community-dwelling stroke survivors to provide feedback to increase motivation and support the continuation of home-based exercise and physical activity. Different feedback options including games, music and lights, are used to increase the motivation of community-dwelling stroke survivors to engage in exercise and physical activity to improve their physical and mental function, increase their walking, and decrease sedentary behaviour, with the ultimate goal of improved participation in society and improved quality of life. Robust and large outcome studies are needed to further investigate the effects of ActivABLES on various outcomes of community-dwelling stroke survivors and caregivers, as well as to examine the cost-effectiveness for the healthcare system.

## Supplementary information

**Additional file 1. ** Appendix I Form for feedback on prototypes during observationsppendix. Appendix II Semi-structured interview guides.

## Data Availability

The prototypes developed are kept at Lund University in Sweden and Aalto University in Helsinki. We do not have a publicly open database. Additional anonymised data from the qualitative interviews and focus groups and further results from the preliminary testing are available upon request from the corresponding author.

## Abbreviations

ADLs: Activities of daily living
CReDECI 2: Criteria for Reporting the Development and Evaluation of Complex Interventions in Healthcare
HCD: Human-centred design
MRC: Medical Research Council
TUI: Tangible user interfaces
