# Supplementary material for: Developing ActivABLES for community-dwelling stroke survivors using the Medical Research Council framework for complex interventions
Source: BMC Health Serv Res. 2020 May 25;20:463. doi: 10.1186/s12913-020-05198-2 (PMC7249380; doi:10.1186/s12913-020-05198-2)
Supplement: Supplementary file 1 — Additional file 1. Appendix I Form for feedback on prototypes during observationsppendix. Appendix II Semi-structured interview guides. [file 12913_2020_5198_MOESM1_ESM.docx]

# Appendices

Appendix I Form for feedback on prototypes during observations

Appendix II Semi-structured interview guides

**Appendix I** Form for feedback on prototypes during observations
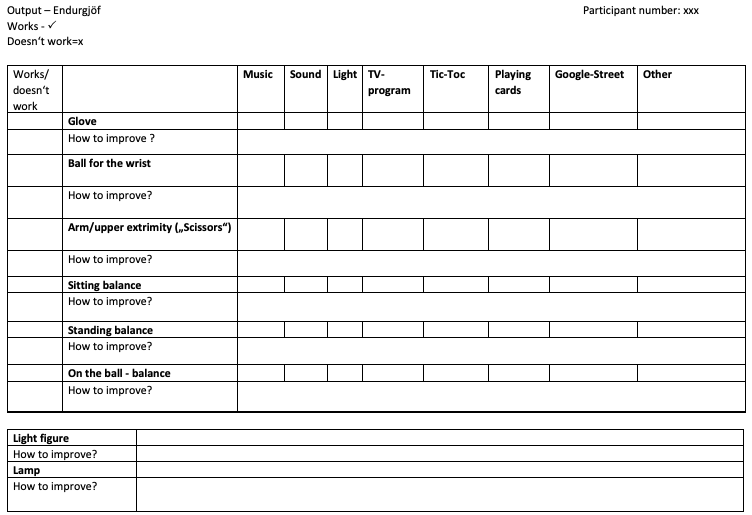


**Appendix II** Semi-structured interview guides

# Preliminary testing of ActivABLES – February 2107

# Semi-structured interview guide

# Stroke survivors

Questions:

1. *Is there something you can´t do but you would like to be able to do?*
2. *Is there something you need to do but is difficult for you?*
3. *Do you exercise by yourself?*
4. *What kind of do you like?*
5. *What kind of exercises do you think are difficult?*
   1. *Why/how are they difficult?*
   2. *What would help you?*
6. *Does your spouse/relative/rehabilitation professional assist or encourage you to exercise?*
7. *What could possible motivate/encourage you to exercise?*
8. *Do you think these tools could motivate/support you to do exercises? – Do you see a purpose to use these tools to maintain or improve you health?*
   1. *Why?*
   2. *Why not?*
9. *Did you liked the exercises/tools ?*
10. *Are the exercises too easy/too difficult for you?*
11. *Do you think these tools could motivate (or not) your spouse/relative to do exercises?*
    1. *Why and how?*
12. *How can we improve these tools? Do you see something we should do differently?*

**Preliminary testing of ActivABLES – February 2107**

**Semi-structured interview guide**

**Caregivers**

Questions:

1. *Does your spouse/relative exercise at home*
2. *Does your spouse/relative exercise by him/herself (own initiative)?*
3. *Do you encourage your spouse/relative to exercise?*
4. *Do you help your spouse/relative to exercise?*
   1. *If you do, how? Can you describe?*
   2. *If you don´t, why not? – Can you describe?*
5. *What kind of exercises does your spouse/relative like to do?*
6. *What kind of exercises does he/she feel are difficult to do?*
   1. *Do you know why/how they are difficult?*
   2. *What would help him/her?*
7. *Do you think these tools could assist your spouse/relative to do exercises?*
   1. *Why?*
   2. *Why not?*
8. *Did you realize what your spouse/relative was supposed to do while using the tools?*
9. *Do you think your spouse/relative liked these exercises ?*
10. *Do you think the exercises were too easy/too difficult for your spouse/relative?*
11. *Do you think these tools could motivate (or not) your spouse/relative to do exercises?*
    1. *Why and how?*
12. *How can we improve these tools? Do you see something we should do differently?*
